# Mood and personality interact to determine cognitive biases in pigs

**DOI:** 10.1098/rsbl.2016.0402

**Published:** 2016-11

**Authors:** Lucy Asher, Mary Friel, Kym Griffin, Lisa M. Collins

**Affiliations:** 1 Centre for Behaviour and Evolution, Institute of Neuroscience, University of Newcastle, Newcastle NE1 7RU, UK; 2 School of Biological Sciences, Queen's University Belfast, Belfast BT7 9BL, UK; 3 School of Life Sciences, University of Lincoln, Lincoln LN6 7DL, UK

**Keywords:** mood, cognitive bias, personality, animal welfare

## Abstract

Cognitive bias has become a popular way to access non-human animal mood, though inconsistent results have been found. In humans, mood and personality interact to determine cognitive bias, but to date, this has not been investigated in non-human animals. Here, we demonstrate for the first time, to the best of our knowledge, in a non-human animal, the domestic pig (*Sus scrofa domesticus*), that mood and personality interact, impacting on judgement. Pigs with a more proactive personality were more likely to respond optimistically to unrewarded ambiguous probes (spatially positioned between locations that were previously rewarded and unrewarded) independent of their housing (or enrichment) conditions. However, optimism/pessimism of reactive pigs in this task was affected by their housing conditions, which are likely to have influenced their mood state. Reactive pigs in the less enriched environment were more pessimistic and those in the more enriched environment, more optimistic. These results suggest that judgement in non-human animals is similar to humans, incorporating aspects of stable personality traits and more transient mood states.

## 1. Introduction

Information processing in humans is known to be pessimistically biased by a negative mood, with a greater expectation of a worse outcome when confronted with ambiguous stimuli [1–4]. By analogy, biases in judgement or cognitive biases have become a popular way to access non-human animal moods [5–8]. Animals in a more positive mood state show ‘optimistic’ biases, characterized by responding to ambiguous stimuli as though they predicted a positive outcome. Conversely, animals in a negative mood show ‘pessimistic’ biases, responding to ambiguous stimuli as if anticipating a negative outcome. If such processes in human and non-human animals operate similarly, then mood is predicted to interact with personality to determine cognitive bias.

Here, we test the hypothesis that mood and personality interact to influence cognitive bias in the domestic pig. The pigs were housed in one of two environments known to influence their mood [9]. Mood, defined as ‘relatively enduring affective states that arise when negative or positive experience in one context or time period alters the individual's threshold for responding to potentially negative or positive events in subsequent contexts or time periods' [10, p. R712], can be affected by the environment [11], with better environments assumed to induce better moods. In contrast, personality is defined as a set of consistent individual differences in behaviour across contexts and time [12]. In pigs, personality is frequently measured, using the coping styles approach [13,14]. Proactivity, at one end of the coping style spectrum, is characterized by more active behavioural responses and less flexible behaviour [15]. Conversely, reactivity indicates more flexible but more passive behaviour. Proactivity/reactivity has been linked to extraversion and neuroticism personality traits in humans. A tendency towards optimism in humans is linked with extraversion, and pessimism with neuroticism [16] and thus may also influence judgement in other animals. We predicted that proactive pigs would respond optimistically in the cognitive bias task, regardless of their housing conditions (and inferred mood state), but reactive pigs would be affected by their housing conditions (and inferred mood state).

## 2. Material and methods

### (a) Animal housing and husbandry

Weaned at four weeks, 36 pigs (commercial crossbreed PIC337 (large white × landrace), *n* = 24 males, *n* = 12 females) were assigned (pseudo-randomly controlling for sex, weight and dam) to either a high- or low-level enriched environment in two groups of 18, replicated three times. Six pigs from each environment and replicate were selected for training. Both environments had solid floors, a slatted area and wooden blocks on chains as enrichment. More enriched environments had deep straw and a larger space allowance (more enriched: 0.62 m^2^ pig^−1^; less enriched: 0.41 m^2^ pig^−1^). Pigs received an ad libitum conventional diet, with artificial lighting 12 h daily and natural light through windows. Ventilation and temperature were automatically controlled (28°C decreasing 0.5°C daily to 19°C). Personality testing occurred at six and eight weeks of age; cognitive bias training and testing was completed by 7–10 weeks of age.

### (b) Cognitive bias testing

**Figure 1.**
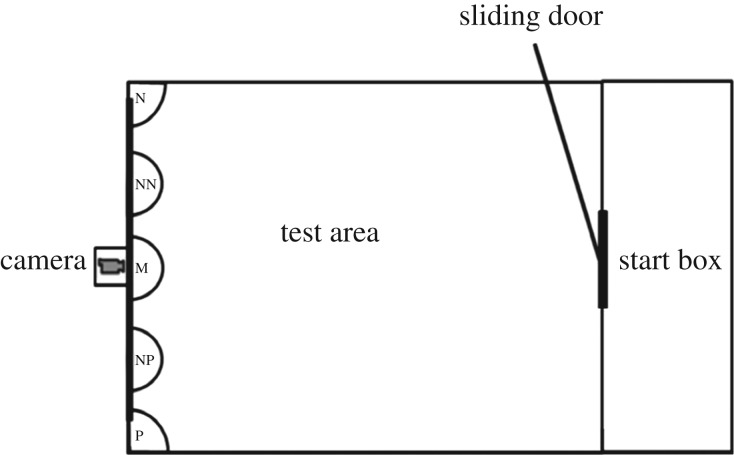
Cognitive bias training and testing arena. N, negative unrewarded location; NN, near negative probe location; M, middle probe location; NP, near positive probe location; P, positive rewarded location.

Pigs were habituated to feeding from a bowl in a test arena (figure 1). After habituation, a false-bottom bowl was used, containing three sugar-coated chocolate sweets and coffee beans to minimize use of olfactory cues. Pigs were trained to associate bowl location with a positive or negative outcome; the positive (P) location in one corner of the arena contained a reward of three sweets, and in the opposite corner, the negative (N) location contained three coffee beans. The location of P/N was pseudo-randomly allocated and counterbalanced over the environmental treatments for each individual. Training progressed from presentations of P, N, P to 5P and 4N in random order, with just one bowl present in each trial. The criteria for learning the task were 80% ‘success’, defined by approaching location P within 30 s and not approaching location N within 30 s. All pigs reached criterion except for nine who failed to habituate (total *n* = 27, 17 male and 10 female; 12 better environment and 15 worse environment). Two tests were conducted per pig, with bowls in three intermediate probe locations (near positive, NP; middle, M; near negative, NN). These were presented in a pseudo-randomized order once per test with the proviso that the M probe was always the first ambiguous probe presented, between ‘recap’ presentations at P and N locations, resulting in nine trials per test (e.g. P, N, M, N, P, NN, P, N, NP). Ambiguous probes were unrewarded but locations P and N contained either sweets or coffee beans, as in training. Pigs were given 30 s to approach the probe after which they were returned to the start box for the next trial. Time to approach the probe was recorded from the point when all four feet were outside the start box.

### (c) Personality testing

For a social isolation (SI) test, pigs were placed individually in a pen (l × w × h: 2.2 × 1.7 × 1.2 m) away from the home environment, where they remained for 3 min without disturbance. After SI test 1, each pig received a 5-min habituation period in the novel object (NO) arena.

On the days following SI tests 1 and 2, pigs participated in an NO test. They were released from a start box (l × w × h: 1 × 1×1.2 m) through a sliding wooden door after 1 min into the arena (l × w × h: 3.6 × 2×1.2 m). A large white bucket and an orange traffic cone were NOs, presented in a pseudo-randomized (across tests, with only one being presented in each test) and counterbalanced (across environments) order. Pigs were given 2 min to enter the arena from the start box. After entering the arena, the door to the start box was closed and an NO was lowered into the middle of the arena on a rope until it was 10 cm from the ground. Once the object was in its final position, the NO test started and lasted for 5 min.

Both test areas and start boxes had plywood walls and concrete floors, which were cleaned between tests and deep cleaned between testing pigs from different pens. Pigs were tested in a randomized order between pens. Within pens, pigs were tested sequentially to minimize disruption to the rest of the pen. Video cameras filmed the tests from above, and duration of standing, exploring, locomotion and line crossing (measure of activity) in both tests was subsequently recorded. In the NO test, latency to contact the object and duration of contact with the NO were also recorded.

### (d) Statistical analysis

To assess personality, repeatability of the behaviours measured in the SI and NO tests was tested using the intraclass correlation coefficient. Proactivity–reactivity (P–R) scores were then calculated from the repeatable behaviours (SI tests: duration standing and exploring; and NO tests: duration of standing, exploring and latency to approach the NO). The P–R scores were calculated as the mean of the *z*-scores of repeatable behavioural measures ([14] has full details of this), with Cronbach's alpha used to measure internal consistency. Only data from the first cognitive bias test were used owing to a decreased latency to approach NP and increased latency to approach NN in test 2 relative to test 1, which was considered evidence of learning such that the probe stimuli may no longer be ambiguous. To standardize for differences in speed of running between individuals, a standardized time to run was created: 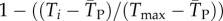 where *T* is time to run, on the *i*th probe trial or to P. 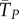 indicates mean time per individual to reach location P and *T*_max_ is maximum time per individual to reach location P. A standardized score of 0 indicates the pig is treating the probe like location N; a score of 1 indicates it is treating the probe like location P. Scores of above 1 are possible if *T_i_* is faster than 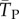.

A linear mixed-effects model with restricted maximum-likelihood was used to analyse log ‘time to run’ as the outcome variable (using lme in nlme package [17]). Individual differences were accounted for as models were weighted by speed of approach to location P, pig and pen identity were included as random effects, P–R scores were covariate and the fixed effects were: treatment (environment), sex and probe location. Fixed effects were dropped if they did not influence model fit, assessed using ANOVA. Sex was dropped from the final model for this reason.

Three *post hoc* mixed-effects models were fitted, one for each probe location. Personality (P–R rank) and environment were included as fixed effects, pen was a random effect and models were weighted by time to approach location P. All analyses were conducted using R [18]. Full analysis, with data and R script are available in electronic supplementary material, S1.

## 3. Results and discussion

We profiled personality in all 36 pigs. High internal reliability (Cronbach's alpha= 0.858) of the repeatable behaviours allowed them to be combined to create the P–R scores for each individual. Lower scores on the P–R index indicate more reactive pigs and higher scores more proactive.

**Figure 2.**
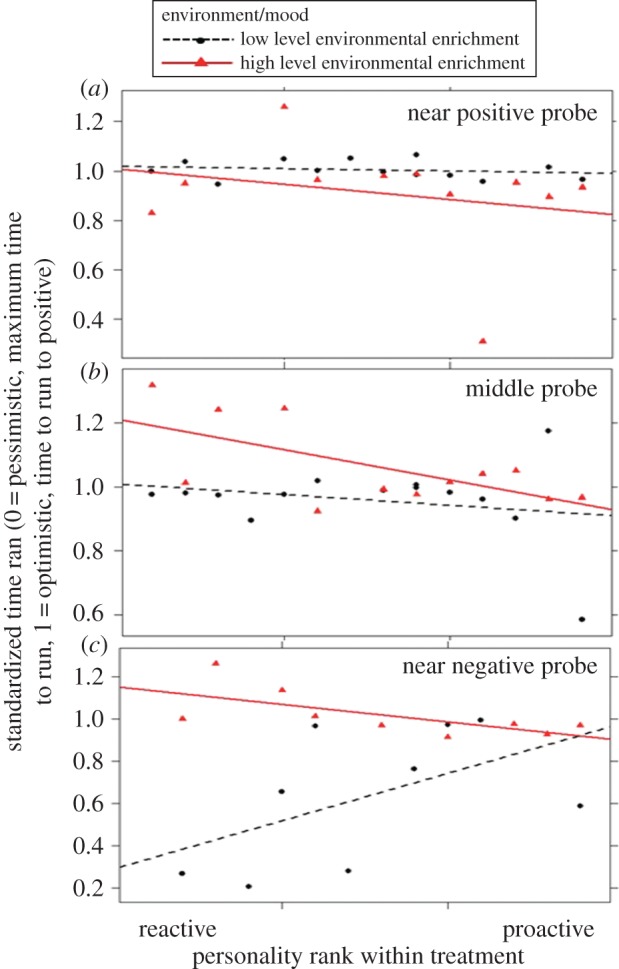
(*a*–*c*) Latency to approach (standardized per individual) unrewarded probes (spatially positioned between locations that were previously rewarded and unrewarded) in a cognitive bias test in more proactive and reactive pigs. Higher standardized time ran scores indicate greater optimism. More proactive personalities were more likely to respond optimistically to unrewarded ambiguous probes. Reactive pigs' optimism/pessimism was affected by their housing conditions. (Online version in colour.)

In humans, information processing biases are dependent on both current mood state and personality [16,17]. Here, we find an analogous effect on cognitive bias in pigs. The speed of approach to the probe locations was significantly affected by an interaction between the location of the probe, personality (rank on P–R scale) and housing environment (which is likely to have affected mood; LMM weighted by individual approach to location P and with pig ID and pen as random effects: *t*_42.7_ =−2.92, *p* = 0.005). Separate analyses on the interaction term revealed that there was no difference between the environments in pigs' speed of approach to the ‘near positive’ probe (LMM weighted by approach to location P and pen as a random effect: *t*_21.8_ = 1.37, *p* = 0.183), or effect of personality (*t*_20.6_ = 0.97, *p* = 0.345, figure 2*a*). To the ‘near negative’ and ‘middle’ probes, there was an interactive effect of environment and personality (near negative: *t*_18_ = 2.38, *p* = 0.028; middle: *t*_23.6_ = 2.40, *p* = 0.025); pigs in the more enriched environment were more optimistic if they were more reactive. However, pigs in the less enriched environment became more pessimistic to the near negative probe if they had a more reactive personality (figure 2*b*,*c*).

Because proactive pigs behaved differently from reactive pigs, these findings could explain some of the inconsistent results between animal cognitive bias tests [19]. Accounting for personality differences between individuals may reduce some of this otherwise unexplained variation, making cognitive bias test outcomes more reliable and robust.

Proactive pigs were less flexible in their response to probes. This fits with existing knowledge about the low flexibility in proactive animals [13]. The reactive pigs were more influenced by their housing environment. Those living in a worse environment were more pessimistic and those in a better environment were more optimistic. Importantly, this finding demonstrates that humans are not unique in combining longer-term personality biases with shorter-term mood biases in judging stimuli [20]. Optimistic and pessimistic responses can both be adaptive depending on the environment [10,21], allowing appropriate responses to reward or threat signals, respectively. The presence of autocorrelated variation in the occurrence of environmental events or in an individual's own state makes fine-scale tuning of responses to cues through the mood system advantageous in comparison with a fixed threshold response system [10]. Therefore, personality and mood jointly influencing an individual's behaviour allows longer-term consistency with shorter-term flexibility for responses to dynamic conditions.

## Supplementary Material

Data set

## Supplementary Material

R code

## Supplementary Material

Output

## Data accessibility

Data, R code and analysis output are available in the electronic supplementary material, S1–S3.
